# Ginsenosides Rb1 and Rg1 Protect Primary Cultured Astrocytes against Oxygen-Glucose Deprivation/Reoxygenation-Induced Injury via Improving Mitochondrial Function

**DOI:** 10.3390/ijms20236086

**Published:** 2019-12-03

**Authors:** Meng Xu, Qing Ma, Chunlan Fan, Xue Chen, Huiming Zhang, Minke Tang

**Affiliations:** 1 Department of Chinese Pharmacology, School of Chinese Materia Medica, Beijing University of Chinese Medicine, Beijing 102488, China; xm_dreaming@163.com (M.X.); maqing988@163.com (Q.M.); 17812003021@163.com (X.C.); minghzhming@163.com (H.Z.); 2 School of Life Sciences, Beijing University of Chinese Medicine, Beijing 102488, China; fanchunlan77@163.com

**Keywords:** ginsenoside Rb1, ginsenoside Rg1, oxygen–glucose deprivation/reoxygenation, astrocytes, reactive oxygen species, oxidative phosphorylation

## Abstract

This study aimed to evaluate whether ginsenosides Rb1 (20-S-protopanaxadiol aglycon) and Rg1 (20-S-protopanaxatriol aglycon) have mitochondrial protective effects against oxygen-glucose deprivation/reoxygenation (OGD/R)-induced injury in primary mouse astrocytes and to explore the mechanisms involved. The OGD/R model was used to mimic the pathological process of cerebral ischemia-reperfusion in vitro. Astrocytes were treated with normal conditions, OGD/R, OGD/R plus Rb1, or OGD/R plus Rg1. Cell viability was measured to evaluate the cytotoxicity of Rb1 and Rg1. Intracellular reactive oxygen species (ROS) and catalase (CAT) were detected to evaluate oxidative stress. The mitochondrial DNA (mtDNA) copy number and mitochondrial membrane potential (MMP) were measured to evaluate mitochondrial function. The activities of the mitochondrial respiratory chain (MRC) complexes I–V and the level of cellular adenosine triphosphate (ATP) were measured to evaluate oxidative phosphorylation (OXPHOS) levels. Cell viability was significantly decreased in the OGD/R group compared to the control group. Rb1 or Rg1 administration significantly increased cell viability. Moreover, OGD/R caused a significant increase in ROS formation and, subsequently, it decreased the activity of CAT and the mtDNA copy number. At the same time, treatment with OGD/R depolarized the MMP in the astrocytes. Rb1 or Rg1 administration reduced ROS production, increased CAT activity, elevated the mtDNA content, and attenuated the MMP depolarization. In addition, Rb1 or Rg1 administration increased the activities of complexes I, II, III, and V and elevated the level of ATP, compared to those in the OGD/R groups. Rb1 and Rg1 have different chemical structures, but exert similar protective effects against astrocyte damage induced by OGD/R. The mechanism may be related to improved efficiency of mitochondrial oxidative phosphorylation and the reduction in ROS production in cultured astrocytes.

## 1. Introduction

Ischemic stroke is one of the leading causes of adult disability in the world. Astrocytes are the most abundant cell type in the central nervous system (CNS) and serve supportive and nutritive roles for neurons. Astrocytes perform several functions that are essential for the formation and maintenance of the blood–brain barrier (BBB), the regulation of brain water and ion homeostasis, and presumably many more processes required for the proper biological function of the CNS [1,2]. The impairment or loss of astrocytes may lead to neuronal dysfunction in the CNS. However, their response to ischemia and their role in neuroprotection after a central nervous system injury are not completely clear. Manipulating the protective functions of astrocytes is thus an important strategy for improving ischemic stroke outcomes.

Brain tissue is vulnerable to the damaging effects of free radicals, and oxidative stress plays an important role in cerebral ischemic pathogenesis. It has been confirmed that oxidative stress leads to the cellular damage of astrocytes [3]. Mitochondria are the powerhouses of the cell because they produce adenosine triphosphate (ATP) by oxidative phosphorylation; they use the electron transport chain (ETC) to generate a proton motive force that maintains the mitochondrial membrane potential (MMP) and is utilized to generate ATP. Mitochondrial DNA (mtDNA) is particularly vulnerable to oxidative damage. Mitochondrial DNA depletion syndromes are generally associated with reduced activities of oxidative phosphorylation (OXPHOS) enzymes. Therefore, it seems that mitochondria are one of the primary targets of astrocytes after cerebral ischemia [4].

**Figure 1:**
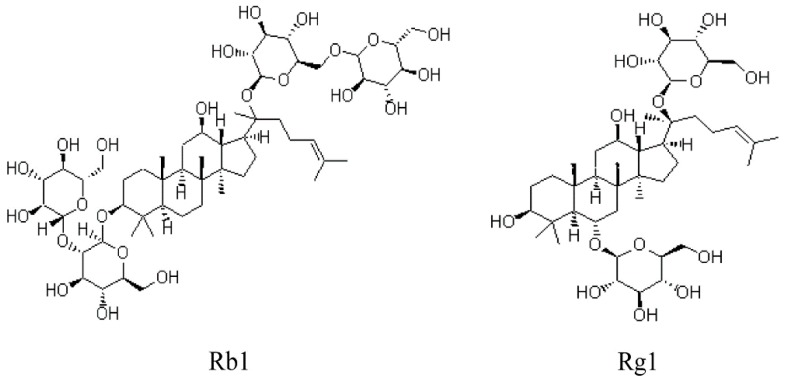
Chemical structures of ginsenoside Rb1 (20-S-protopanaxadiol aglycon) and ginsenoside Rg1 (20-S-protopanaxatriol aglycon).

*Panax ginseng* C.A. Meyer is a commonly used Chinese medicinal herb. Ginsenosides are the primary active components of this herb, and have a variety of pharmacological effects, such as antioxidant, anti-inflammatory, antiapoptotic and neuroprotective properties. Ginsenosides are derivatives of triterpenoid dammarane, which consists of thirty carbon atoms. They can be mainly classified into protopanaxadiol (PD) and protopanaxatriol (PT) ginsenosides, based on the presence of carbohydrate moieties at the C3 or C6 position [5]. Ginsenoside Rb1 (20-S-protopanaxadiol aglycon) and ginsenoside Rg1 (20-S-protopanaxatriol aglycon) are the main active ingredients of *Panax ginseng*. The chemical structures of Rb1 and Rg1 are depicted in Figure 1. Different aglycone structures and different positions of sugar moieties may result in similarities and/or differences in pharmacology and mechanism [6].

An increasing amount of evidence has indicated that Rb1 and Rg1 exert neuroprotective effects both in vivo and in vitro [7,8,9,10,11]. It has been found that Rb1 and Rg1 improve mitochondrial function and regulate the level of ATP to protect nerve cells against cerebral ischemia-reperfusion injury [12,13]. Whether Rb1 and Rg1 have any effects on mitochondrial function under oxygen–glucose deprivation (OGD) and reoxygenation (OGD/R) conditions in astrocytes is still unknown. Whether these two types of saponins show similarities and/or differences in pharmacology under OGD/R conditions remains unclear. Hence, in the present study, we investigated the effects of Rb1 and Rg1 on OGD/R-induced injury and further explored the underlying molecular mechanism in primary cultured astrocytes.

## 2. Results

### 2.1. Validation of Mouse Astrocytes

**Figure 2:**
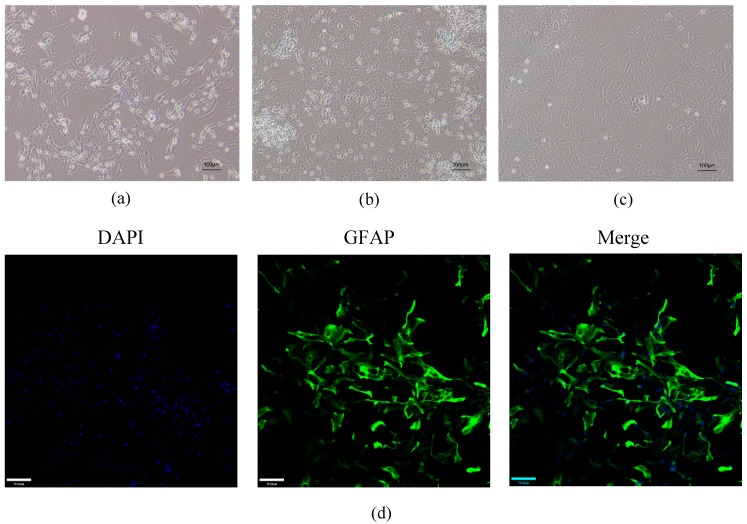
Morphology and immunofluorescence staining of astrocytes. (**a**) The cells had a star-shaped morphology with irregular protrusions after 3 days in the culture. (**b**) After 7 days, there were many protrusions on the surface of the astrocytes. (**c**) Pure astrocyte cultures were obtained after subculture. (**a**–**c**) Scale bar: 100 µm. (**d**) Immunocytochemical staining using green fluorescent dye showed that the astrocytes expressed glial fibrillary acidic protein (GFAP). All nuclei were stained with 4′,6-diamidino-2-phenylindole (DAPI) (blue fluorescent dye). Scale bar: 70 µm.

After 3 days in culture, cells had a star-shaped morphology with irregular protrusions (Figure 2a). An increase in cell volume was observed after 7 days in culture. There were many cell protrusions on the surface of the astrocytes (Figure 2b). Pure astrocyte cultures were obtained after subculture (Figure 2c). The purity of the cultures was verified by immunofluorescence staining for glial fibrillary acidic protein (GFAP), a known marker for astrocytes. All cells in culture appeared to express high levels of GFAP (Figure 2d).

### 2.2. Rb1 and Rg1 Attenuated OGD/R-Induced Injury in Cultured Astrocyte

**Figure 3:**
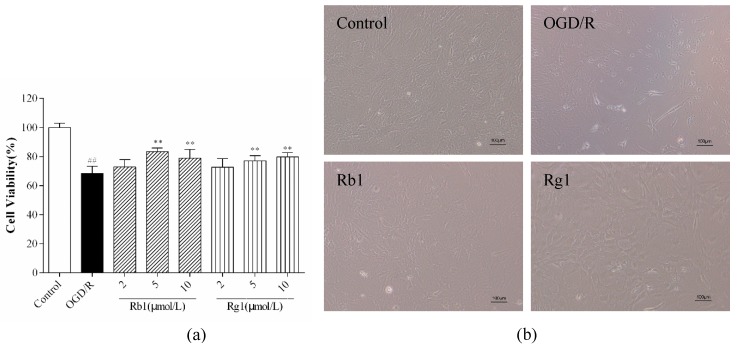
Rb1 and Rg1 increased cell viability in oxygen-glucose deprivation/reoxygenation (OGD/R)-treated astrocytes. After 6 h of OGD, astrocytes were reoxygenated for 24 h. OGD/R-treated astrocytes were incubated with varying concentrations of Rb1 or Rg1 (2, 5, and 10 µM). (**a**) The CCK-8 assay was used to examine cell viability. (**b**) The morphology of astrocytes was observed by using an inverted microscope. The results showed that Rb1 (5 µM) and Rg1 (10 µM) significantly increased cell viability and improved cell state. The data are expressed as the mean ± SD (*n* = 8). ^##^ *p* < 0.01 and ^#^ *p* < 0.05 versus control cells; ** *p* < 0.01 and * *p* < 0.05 versus OGD/R-treated cells. Scale bar: 100 µm.

As shown in Figure 3a, we investigated cell viability under different experimental conditions. Compared with that of control cells, cell viability was significantly decreased in the cells that were injured by OGD/R (*p* < 0.01). After 6 h of OGD, astrocytes were reoxygenated for 24 h, and Rb1 and Rg1 at concentrations of 5 and 10 µM significantly increased cell viability (*p* < 0.01). Based on these findings, 5 µM Rb1 and 10 µM Rg1 were selected as the final treatment concentrations. As shown in Figure 3b, control astrocytes grew well and had good refraction. After OGD/R treatment, the astrocyte refraction was weakened, the protrusions became shorter or disappeared, and the cells detached. The administration of Rb1 (5 µM) and Rg1 (10 µM) significantly improved cell state and enhanced cell refraction and the relationship between cell protrusions. These data suggest that Rb1 and Rg1 may protect astrocytes from OGD/R-induced damage.

### 2.3. Rb1 and Rg1 Suppressed ROS Production and Increased CAT Activity in OGD/R-Treated Astrocytes

**Figure 4:**
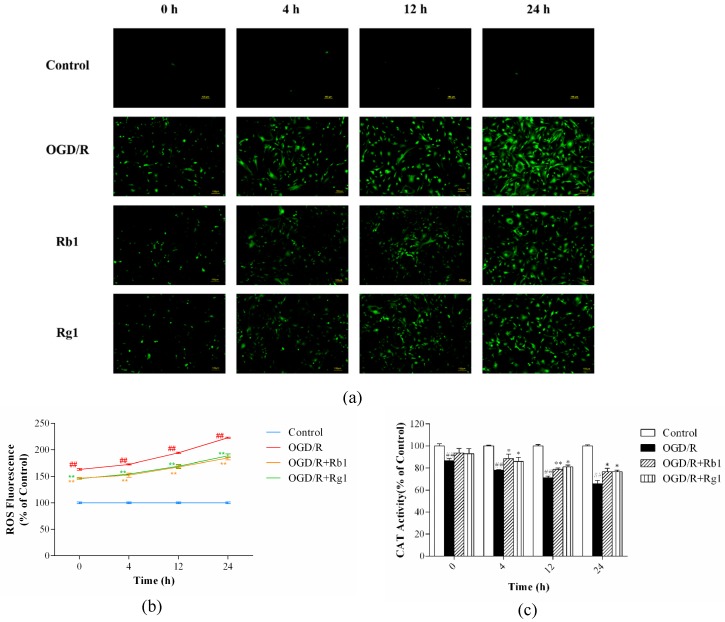
Rb1 and Rg1 decreased reactive oxygen species (ROS) production and increased catalase (CAT) activity in oxygen-glucose deprivation/reoxygenation (OGD/R)-treated astrocytes. Astrocytes were incubated with Rb1 (5 µM) or Rg1 (10 µM). After 6 h of OGD, astrocytes were reoxygenated for 0, 4, 12, or 24 h. (**a**) A 2′,7′-dichlorodihydrofluorescein diacetate (DCFH-DA) probe was used to monitor ROS production. (**b**) Fluorescence images were analyzed using ImageJ software. (**c**) Cells were collected to measure CAT activity. The results showed that Rb1 (5 µM) and Rg1 (10 µM) led to decreases in ROS production and significant increases in CAT activity. The values are expressed as the mean ± SD (*n* = 3). ^##^ *p* < 0.01 and ^#^ *p* < 0.05 versus control cells; ** *p* < 0.01 and * *p* < 0.05 versus OGD/R-treated cells. Scale bar: 100 µm.

Oxidative stress is caused by a net imbalance between the pro-oxidants and the antioxidants in the cell, leading to excessive ROS levels that damage all biomolecules [14]. CAT is a major enzyme for the degradation of ROS [15]. Compared with that in control cells, the production of intracellular ROS was significantly enhanced in OGD/R-treated astrocytes (*p* < 0.01) (Figure 4a,b), but CAT activity was decreased significantly (*p* < 0.01) (Figure 4c). Rb1 (5 µM) and Rg1 (10 µM) administration significantly reduced the production of ROS (*p* < 0.01) and increased CAT activity (*p* < 0.01) compared to the levels in OGD/R-treated astrocytes. These results suggest that Rb1 and Rg1 reduce the production of ROS and increase CAT activity in OGD/R-treated astrocytes.

### 2.4. Rb1 and Rg1 Inhibited MMP Depolarization and Increased mtDNA Content in OGD/R-Treated Astrocytes

**Figure 5:**
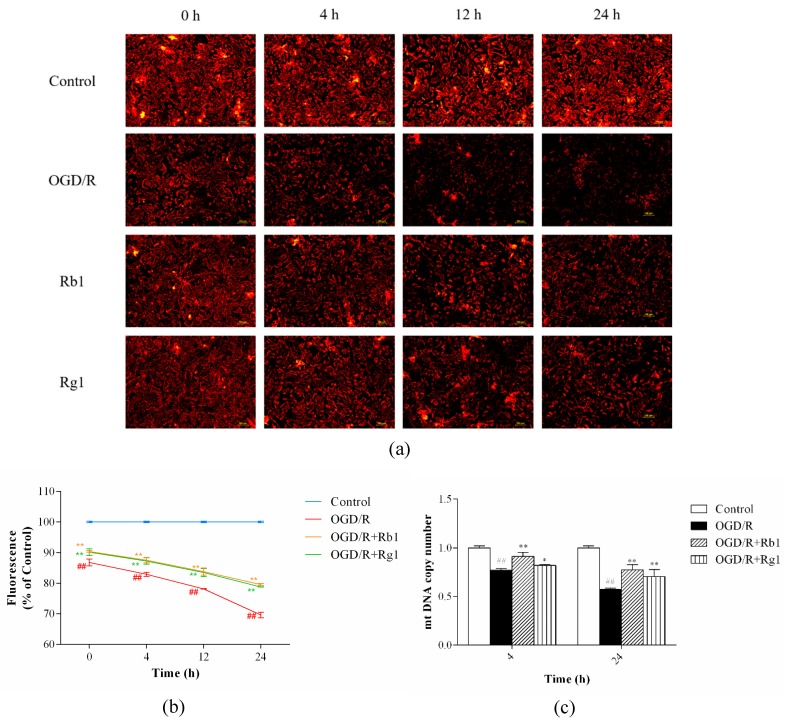
Rb1 and Rg1 attenuated the mitochondrial membrane potential (MMP) depolarization and increased the mitochondrial DNA (mtDNA) content in oxygen-glucose deprivation/reoxygenation (OGD/R)-treated astrocytes. After 6 h of OGD, astrocytes were reoxygenated for 0, 4, 12, or 24 h. (**a**,**b**) Cells were collected to detect the MMP. After 6 h of OGD, astrocytes were reoxygenated for 4 or 24 h. (**c**) Cells were collected to examine the mtDNA copy number by real-time quantitative PCR. Rb1 (5 µM) and Rg1 (10 µM) administration attenuated the MMP depolarization and increased the mtDNA content. The values are expressed as the mean ± SD (*n* = 3). ^##^ *p* < 0.01 and ^#^ *p* < 0.05 versus control cells; ** *p* < 0.01 and * *p* < 0.05 versus OGD/R-treated cells. Scale bar: 100 µm.

ROS can affect mtDNA, causing modulation of the synthesis of ETC components, decreased ATP production, depolarized MMP, and increased leakage of ROS [16,17]. After 6 h of OGD, astrocytes were reoxygenated for 0, 4, 12 or 24 h; compared to the control cells, MMP depolarization was continuously enhanced in OGD/R-treated astrocytes (*p* < 0.01) (Figure 5a,b). After 6 h of OGD, astrocytes were reoxygenated for 4 or 24 h; compared with that in the control cells, OGD/R-induced mtDNA content was decreased in astrocytes (*p* < 0.01) (Figure 5c). Rb1 (5 µM) and Rg1 (10 µM) administration significantly attenuated the MMP depolarization (*p* < 0.01)) and increased mtDNA copy number (*p* < 0.01), compared to that in OGD/R-treated astrocytes. These data suggest that Rb1 and Rg1 protect mitochondrial function against OGD/R-induced injury in astrocytes.

### 2.5. Rb1 and Rg1 Increased the Activities of Mitochondrial Respiratory Chain Complexes I–V and ATP Levels

**Figure 6:**
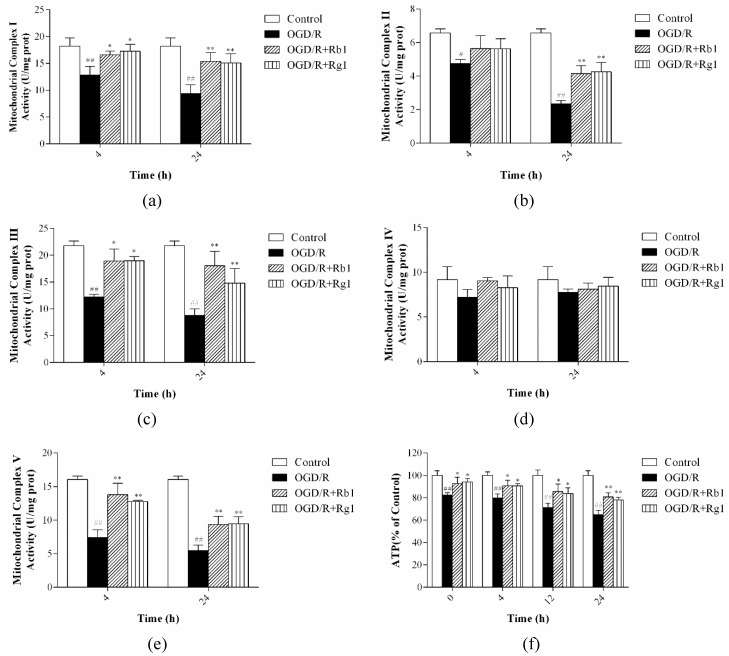
Rb1 and Rg1 regulated the level of oxidative phosphorylation (OXPHOS) in oxygen-glucose deprivation/reoxygenation (OGD/R)-treated astrocytes. Astrocytes were incubated with Rb1 (5 µM) or Rg1 (10 µM). (**a**–**e**) After 6 h of OGD, astrocytes were reoxygenated for 4 or 24 h, and cells were collected to detect the activities of complexes I-V. (**f**) After 6 h of OGD, astrocytes were reoxygenated for 0, 4, 12, or 24 h, and cells were collected to measure the level of ATP. The results revealed that Rb1 (5 µM) and Rg1 (10 µM) administration increased the activities of complexes I, II, III, and V and ATP levels, but there was no significant difference in complex IV activity among the experimental groups. The values are expressed as the mean ± SD (*n* = 3). ^##^ *p* < 0.01 and ^#^ *p* < 0.05 versus control cells; ** *p* < 0.01 and * *p* < 0.05 versus OGD/R-treated cells.

Most pathogenic mtDNA mutations induce defects in mitochondrial oxidative phosphorylation [18]. The mitochondrial respiratory chain (MRC) consists of five multimeric protein complexes (I–V) that drive the production of ATP. After 6 h of OGD, astrocytes were reoxygenated for 4 or 24 h. Compared with those in control cells, the activities of complexes I, II, III, and V were significantly decreased in OGD/R-treated astrocytes (*p* < 0.01) (Figure 6a–c,e). After 6 h of OGD, astrocytes were reoxygenated for 0, 4, 12, or 24 h, and the level of ATP was further decreased after OGD/R-induced injury compared to that in control cells (*p* < 0.05) (Figure 6f). Incubation with Rb1 (5 µM) and Rg1 (10 µM) increased the activities of complexes I, II, III, and V (*p* < 0.05) and ATP levels (*p* < 0.05), but there was no significant difference in complex IV activity among the experimental groups (Figure 6d). These data suggest that Rb1 and Rg1 reverse the impairment of oxidative phosphorylation after OGD/R-induced damage in astrocytes.

## 3. Discussion

In this study, we demonstrated that Rb1 and Rg1, the main ingredients of ginseng, reduced oxidative stress and restored OXPHOS function in OGD/R-treated astrocytes. These results indicate that Rb1 and Rg1 have different chemical structures but exert similar protective effects against astrocyte damage induced by OGD/R. The mechanism may be related to improved OXPHOS function and reduced ROS production in cultured astrocytes. Astrocytes are the most abundant cell type in the central nervous system (CNS), and serve neurons in supportive and nutritive roles. Several reports have shown that the impairment or loss of astrocytes contributes to neuronal dysfunction in the CNS [19,20,21]. When assayed in culture, the exposure of rat or mouse astrocytes to OGD/R causes dysfunction, which is reflected by altered cellular activities [22,23,24]. Here, we developed a cell-based model using cultured astrocytes that were exposed to OGD/R and confirmed the detrimental effect of OGD/R exposure. Meanwhile, coincubation with Rb1 and Rg1 improved astrocyte viability, indicating that Rb1 and Rg1 protect astrocytes against OGD/R-induced injury.

Oxidative stress plays a critical role in cell death, which occurs under diverse neuropathological conditions. Several studies have reported that OGD/R mediates excessive ROS production and oxidative stress, which can lead to nerve and glial cell death [25,26]. During the phase of ischemia and reperfusion, antioxidant capacity is limited by the consumption of neutralizing scavenging antioxidants or excessive oxidative stress. The sources of cellular ROS include mitochondria and nicotinamide adenine dinucleotide phosphate (NADPH) oxidases (NOXs), in which mitochondria are the main source [27]. It was reported that the excess ROS (mitochondrial ROS or intracellular ROS) can cause oxidative stress, damage mtDNA, open mitochondrial permeability transition pores, depolarize MMP, and result in mitochondrial dysfunction [28,29]. CAT, the primary enzyme responsible for protecting the cell from reactive oxygen species, is present in the peroxisomes of cells. Therefore, in this study, we selected intracellular ROS production and CAT to evaluate intracellular oxidative stress. Our results revealed that intracellular ROS were significantly increased and that CAT activity was markedly reduced when astrocytes were exposed to OGD/R conditions. Rb1 or Rg1 administration reduced the OGD/R-induced intracellular ROS production and caused a significant enhancement of CAT activity. These data suggest that OGD/R-induced ROS production exceeds the natural antioxidant capacity and leads to oxidative stress, and that Rb1 and Rg1 may exert their protective effect on OGD/R-treated astrocytes by reducing the ROS overload and increasing CAT activity. Mitochondrial reactive oxygen species (mitochondrial ROS) production is a tightly regulated redox signal that transmits information from the organelle to the cell. However, the exact mechanism of mitochondrial ROS production, after Rb1 and Rg1 stimulation, is not clearly understood. Furthermore, the regulating mechanisms responsible for maintaining the redox state are not yet fully known. Therefore, further studies are necessary to elucidate the mechanisms by which the mitochondrial ROS-mediated redox signaling pathway is modulated by Rb1 and Rg1 in OGD/R conditions.

Oxidative stress can promote mitochondrial permeability transition pore (MPTP) opening, change the mitochondrial membrane potential, and disrupt the mitochondrial respiratory chain, ultimately resulting in apoptosis or cell death [30]. The disruption of the mitochondrial membrane potential is a hallmark and a key step in the activation of the mitochondrial pathway. ROS cause oxidative damage to biological molecules, and the accumulated damage further promotes cell damage in oxidative stress. The excess ROS caused DNA damage. However, mtDNA is a naked circular double-stranded DNA, which lacks histones, and its repair capacity is weaker than nuclear DNA [31,32]. Therefore, mtDNA is more sensitive to endogenous oxidative damage than nuclear DNA [33]. Here, our study showed that OGD/R increased the depolarization of the MMP and decreased the mtDNA copy number. Rb1 or Rg1 administration attenuated OGD/R-induced MMP depolarization and increased the mtDNA content. These data demonstrate that Rb1 and Rg1 improve mitochondrial function to protect astrocytes from OGD/R-induced oxidative damage. Also, 8-dihydrodeoxyguanine (8-oxoG) is a specific marker of oxidative damage to DNA [34]. Therefore, it is interesting to investigate the level of DNA damage and the expression of DNA repair enzymes during ischemia and reperfusion. Based on this, further study was designed to investigate the relationship between DNA damage and apoptosis in astrocytes.

ROS can affect mitochondrial DNA (mtDNA), causing modulation of the synthesis of the electron transport chain components, decreasing ATP production, and increasing ROS leakage. Mitochondrial function can be evaluated by analyzing the activities of the individual respiratory chain complexes [35,36,37]. The mitochondrial respiratory chain, also known as the electron transport chain, is crucial to life, and energy production in the form of ATP is the primary mitochondrial function. The mitochondrial respiratory chain, also known as the electron transport chain, is responsible for the coupling of oxidative phosphorylation to energy production. The respiratory chain comprises five multicomponent complexes, namely complex I (NADH dehydrogenase), complex II (succinate dehydrogenase), complex III (cytochrome bc1 complex), complex IV (cytochrome c oxidase), and ATP synthase, also known as complex V, which act as a series of electron carriers of graded redox midpoint potentials that can undergo alternative oxidation and reduction [38]. This gradient is subsequently utilized in the production of high energy phosphates. This study showed that the activities of MRC complexes I, II, III, and V, and the level of ATP, were significantly decreased after OGD/R exposure. However, no significant differences were observed in the activity of complex IV between the control and the OGD/R groups. Rb1 or Rg1 administration significantly increased the activities of the complexes and the ATP levels in astrocytes under OGD/R conditions. Therefore, it is reasonable to conclude that reducing oxidative stress and increasing the activities of respiratory chain enzymes improves the efficiency of mitochondrial oxidative phosphorylation and may be the main protective mechanism of Rb1 and Rg1 in astrocytes.

In recent decades, with the continuous progress of technology, research on ginseng has made great progress. Approximately 40 ginsenosides have been identified from the root of ginseng [39,40]. Ginsenosides Rb1 and Rg1 are regarded as the main compounds responsible for many pharmaceutical actions of ginseng. Because ginsenosides produce different effects, and because a single ginsenoside initiates multiple actions in the same tissue, the overall pharmacology of ginseng is complicated. In this study, both Rg1 and Rb1 showed similar protective effects in reducing ROS production and improving mitochondrial oxidative phosphorylation capacity in OGD/R-treated astrocytes. In addition, it was reported that astrocytes have a strong tolerance in an ischemic state and can protect neurons by reducing the release of excitatory amino acids, regulating energy metabolism, inhibiting oxidative stress, and secreting neurotrophic factors [41]. Compared to other cell types, neurons are more dependent on mitochondrial oxidative phosphorylation (OXPHOS) to fulfill their energy demands. This is because neurons have a limited capacity to upregulate glycolysis or to counteract oxidative damage under injury (neurotoxicity and ischemia) conditions [42]. Moreover, previous studies have reported that the signal transducer and activator of transcription (STAT3) pathway plays significant regulatory roles in maintaining mitochondrial function in myocardial ischemia [43,44]. However, there is little research about mitochondrial STAT3 in cerebral ischemia-reperfusion injury. Moreover, it remains unclear whether the effects of mitochondrial STAT3 on OXPHOS in astrocytes and the protective effects of Rb1 and Rg1 on astrocyte OXPHOS levels have an effect on neuronal viability under OGD/R conditions. Therefore, it is necessary to conduct further investigations aimed at the effects of Rb1 and Rg1 in the regulation of mitochondria function and their associated signaling pathway in neurons and astrocytes cocultured under OGD/R-induced damage by measuring experimental indexes including OXPHOS, mitochondrial function, cell redox state, and STAT3 relevant signaling pathway.

## 4. Materials and Methods

### 4.1. Animals

Institute of Cancer Research (ICR) mice (within 24 h after birth) were purchased from Vital River Laboratory Animal Technology Co., Ltd. in Beijing, China (certificate number, SCXK [Jing] 2016–0006). All of the experiments involving animals were carried out in accordance with the National Institutes of Health Guide for Care and Use of Laboratory Animals [45], and the Institutional Animal Care and Use Committee of Beijing University of Chinese Medicine approved the protocol (BUCM-4-2018050701-2065, 07 May 2018). All efforts were made to minimize animal suffering and reduce the number of animals used.

### 4.2. Cell Culture

The astrocyte cultures were prepared according to a previously described method, but with slight modifications [46]. Mice were sterilized with 75% alcohol. The cerebral cortex was removed from the skull, and the meninges were carefully stripped away. The cerebral tissues were cut into small pieces and digested with 0.25% trypsin (Corning Inc., Corning, NY, USA) for 15 min; these tissues were filtered through a 100 µm cell strainer. After centrifugation at 1000× rpm for 5 min, the pellets were suspended in an astrocyte medium (AM, ScienCell Research Laboratories, Carlsbad, CA, USA). The above suspension was transferred to a 25 cm^2^ flask which was precoated with 0.01% poly-L-lysine (PLL, Biodee Biotechnology, Beijing, China) and cultured at 37 °C in a 5% CO_2_ humidified incubator (Thermo Fisher Scientific, Waltham, MA, USA). The suspension was preadhered in an uncoated cell culture flask for 30 min at 37 °C to remove the fibroblasts. The culture medium was replaced with a fresh medium every 2–3 days. The confluent cultures were passaged using 0.25% trypsin to dissociate the cells at a split ratio of 1:2.

### 4.3. Identification of Astrocytes

The astrocytes were identified by immunofluorescence staining for the expression of glial fibrillary acidic protein (GFAP). Briefly, cells were cultured at a density of 1 × 10^5^ cells/mL in PLL-coated coverslips. After the cells attached to the flask, immunofluorescence staining was used to identify the astrocytes. Cells were fixed with freshly prepared 4% paraformaldehyde (Solarbio, Beijing, China) for 30 min at 4 °C. After fixation, the cells were washed three times with phosphate buffer solution (PBS, containing 5% penicillin/streptomycin, Corning Inc., Corning, NY, USA) and lysed with 0.1% Triton X-100 (Solarbio) for 10 min at room temperature. Then, the cells were washed three times with PBS, blocked with 10% normal goat serum (Solarbio) for 30 min, and incubated with a primary antibody (rabbit anti-GFAP, 1:200; ab7260, Abcam, Cambridge, UK) in a moist chamber at 4 °C overnight. After three washes with PBS, the cells were incubated for 1 h at 37 °C in the dark with fluorescein isothiocyanate (FITC)-labeled goat anti-rabbit secondary antibody (1:200; Solarbio), and stained with 4′,6-diamidino-2-phenylindole (DAPI, Cell Signaling Technology, Danvers, MA, USA). All images were captured by using a fluorescence microscope with appropriate filters (Nikon Corporation, Tokyo, Japan).

### 4.4. OGD/R Procedure and Drug Treatment

Oxygen–glucose deprivation and reoxygenation (OGD/R) was used to mimic the pathological process of ischemia-reperfusion in vivo [47]. The astrocytes were washed twice with prewarmed PBS and incubated in a medium without glucose to establish OGD conditions. The astrocytes were then exposed to 95% N_2_ and 5% CO_2_ in a hypoxic humidified incubator for 6 h, and the medium was incubated with AM under normoxic conditions for 0, 4, 12, or 24 h to establish OGD/R. Cells cultured in growth culture medium under normoxic conditions served as a control. Rb1 and Rg1 (purity > 98%, Yuanye Biotechnology, Shanghai, China) were applied to the cell cultures (medium with no glucose or AM) during OGD/R injury. Astrocytes were investigated at 0, 4, 12, or 24 h after OGD/R.

### 4.5. CCK-8 Assay

Astrocytes were plated at a density of 1 × 10^5^ cells/mL on PLL-coated 96-well plates with 100 µL culture medium per well and exposed to experimental conditions. Cultured astrocytes were treated with varying concentrations of Rb1 (2, 5, 10 µM) or Rg1 (2, 5, 10 µM). After the cells were exposed to 24 h of OGD/R, 10-µL CCK-8 solution (Dojindo Laboratories, Kyushu, Japan) was added to each culture well. The optical density (OD) value at the wavelength of 450 nm was read using a microplate reader (Thermo Fisher Scientific, Waltham, MA, USA).

### 4.6. Morphology Observation of Astrocytes

Astrocytes were plated at a density of 1 × 10^5^ cells/mL on PLL-coated 6-well plates with 2.5 mL culture medium per well and exposed to experimental conditions. Cultured astrocytes were treated with varying concentrations of Rb1 (5 µM) or Rg1 (10 µM). After the cells were exposed to 24 h of OGD/R, all images were captured by using an inverted microscope (Nikon Corporation, Tokyo, Japan).

### 4.7. Measurement of Intracellular ROS Levels

The intracellular ROS levels were measured with the ROS Activity Assay Kit (Nanjing Jiancheng Bioengineering Institute, Nanjing, China) according to the manufacturer’s instructions. Briefly, the astrocytes were plated at a density of 1 × 10^5^ cells/mL on PLL-coated 24-well plates with 1 mL culture medium per well and exposed to experimental conditions. After cells were exposed to 0, 4, 12, or 24 h of OGD/R, 2′,7′-dichlorodihydrofluorescein diacetate (DCFH-DA)-loaded cells were observed under a fluorescence microscope (Nikon Corporation, Tokyo, Japan). Confocal images were processed with ImageJ software (National Institutes of Health, Bethesda, MD, USA).

### 4.8. Catalase Activity

Astrocytes were grown at a density of 1 × 10^5^ cells/mL in a 25 cm^2^ culture flask coated with 0.01% PLL in a total volume of 5 mL culture medium and exposed to experimental conditions. After Rb1/Rg1 treatment and OGD/R exposure, cell extracts were collected for measurement of CAT activity using a catalase analysis kit (Beyotime Biotechnology, Shanghai, China) according to the manufacturer’s instructions. Protein concentration was measured using the BCA Protein Quantitation Assay Kit (KeyGen Biotech, Jiangsu, China).

### 4.9. Detection of the Mitochondrial Membrane Potential

The mitochondrial membrane potential was measured using a JC-1 dye kit (KeyGen Biotech, Jiangsu, China). As previously described [48], astrocytes were seeded at a density of 1 × 10^5^ cells/mL on PLL-coated 24-well plates. After Rb1/Rg1 treatment and OGD/R exposure, the cells were incubated in the dark with JC-1 for 20 min at 37 °C, and images were taken with a fluorescence microscope (Nikon Corporation, Tokyo, Japan).

### 4.10. Measurement of Intracellular ATP Levels

Astrocytes were seeded at a density of 1 × 10^5^ cells/mL in a 25 cm^2^ culture flask coated with 0.01% PLL and exposed to experimental conditions. After Rb1/Rg1 treatment and OGD/R exposure, cells were collected to measure cellular ATP levels using the ATP Detection Assay Kit (Beyotime Biotechnology, Shanghai, China) according to the manufacturer’s instructions. Briefly, cells were harvested and lysed in the lysis buffer provided with the kit, and the RLU value was measured by a luminometer (Molecular Devices, San Jose, AZ, USA). Protein concentration was measured using the BCA Protein Quantitation Assay Kit (KeyGen Biotech, Jiangsu, China).

### 4.11. Real-Time Quantitative Polymerase Chain Reaction (PCR) Analysis

DNA was extracted from the astrocytes using the HiPure Tissue DNA Kit (Magen Biotech, Guangzhou, China). DNA quantity and purity were determined. The OD260/OD280 ratios of the isolated DNA were within the range of 1.8–2.0. The forward and reverse primers used to amplify β-actin were 5′-TGTTACCAACTGGGACGACA-3′ and 5′-CTATGGGAGAACGGCAGAAG-3′, respectively. The forward and reverse primers used to amplify mtDNA were 5′-AACATACGAAAAACACACCCATT-3′ and 5′-AGTGTATGGCTAAGAAAAGACCTG-3′, respectively. The amplification was performed in 96-well plates on the StepOnePlus Real-Time System (Thermo Fisher Scientific). The PCR mixture consisted of DNA samples mixed with primers and SYBR Green Real Master Mix (Thermo Fisher Scientific) in a final volume of 20 µL. The cycling conditions were as follows: initial denaturation at 95 °C for 10 min; amplification for 40 cycles, including denaturation at 95 °C for 15 s and annealing at 60 °C for 60 s. All samples were run in triplicate, and the average values were calculated. The relative mtDNA copy number was normalized to a single-copy nuclear β-actin gene, and the relative mtDNA copy number was calculated using the 2^−ΔΔC*t*^ method [49,50].

### 4.12. Isolation of Mitochondria from Astrocytes

Mitochondria were isolated from astrocytes using a cell mitochondrial kit (Beyotime Biotechnology, Shanghai, China) in accordance with the manufacturer’s instructions. Briefly, the cells (at least 5 × 10^6^ cells) were harvested and washed twice with ice-cold PBS. Then, they were incubated with cell lysis buffer at 4 °C and homogenized with a glass homogenizer. The cell lysate was centrifuged at 600× *g* for 10 min to remove unbroken cells, and then the supernatant was centrifuged at 11,000× *g* for 15 min at 4 °C. The resulting pellet, which contained the mitochondrial fraction, was collected. The mitochondrial pellet was resuspended in mitochondrial lysis buffer and stored at 4 °C for immediate use or at −80 °C for later use.

### 4.13. Assay of the Activities of Mitochondrial Respiratory Chain Complexes I–V in Astrocytes

Mitochondria were isolated from astrocytes to measure the activities of the mitochondrial respiratory chain complexes I–V. The activities of the complexes I–V were detected with mitochondrial respiratory chain complexes I, II, III, IV, and V kits (Solarbio, Beijing, China), according to the manufacturer’s instructions. Briefly, the mitochondria were dissolved in an appropriate mitochondrial lysis buffer and disrupted by sonication (power 20%, ultrasound 5 s, interval 10 s, repeat 15 times). Complex I activity assay: according to the manufacturer’s instruction, the sample, the reagent 1, the working solution and the reagent 4 were sequentially added to the 96-well plate, and then rapidly mixed; the OD_1_ value at the 10th second was measured at 340 nm. After that, the 96-well plate was reacted at 37 °C for 2 min; the OD_2_ value at the 2nd minute was recorded. Complex II activity assay: the sample, the reagent 6, and the working solution were sequentially added to the 96-well plate and then rapidly mixed; the initial OD_1_ value and the 2nd minute OD_2_ value were recorded at 605 nm. Complex III activity assay: the reagent 3 and the working solution were added to the 96-well plate and reacted at 37 °C for 2 min, while continuing to add the sample and the distilled water, and recording the initial OD_1_ value and the 2nd minute OD_2_ value at 550 nm. Complex IV activity assay: the sample, the distilled water, and the working solution were sequentially added to the 96-well plate and rapidly mixed, then the initial OD_1_ value and the 2nd minute OD_2_ value at 550 nm were recorded. Complex V activity assay: the reagent 2, the reagent 3, and the sample were sequentially added to the 1.5 mL centrifuge tube and reacted at 37 °C for 30 min, then the reagent 4 was added to the reactive system and rapidly mixed. The system was centrifuged at 8000× rpm for 10 min. The phosphorus reagent was added to the supernatant and reacted at 40 °C for 10 min; the OD value was measured at 660 nm. The activities of mitochondrial complexes I–V were calculated according to the formula of the manufacturer’s instructions. The mitochondria protein concentration was measured using the BCA Protein Quantitation Assay Kit (KeyGen Biotech, Jiangsu, China).

### 4.14. Statistical Analysis

All data are shown as the mean ± SD. One-way analysis of variance followed by an LSD test was used to analyze all the data. Values of *p* < 0.05 were considered significant. Statistical analyses were performed using SAS 9.4 software (SAS Inc., Raleigh, NC, USA).

## 5. Conclusions

In conclusion, our findings indicate that Rb1 and Rg1 are endowed with a significant protective mitochondrial function against OGD/R-induced damage in astrocytes. The mechanism may be related to improved efficiency of mitochondrial oxidative phosphorylation and reduced ROS production. However, further research will rely heavily on the use of cocultured neurons and astrocytes to elucidate the effects of Rb1 and Rg1 in the regulation of mitochondria function and their associated signaling pathway under OGD/R conditions.

## Abbreviations

Rb1	Ginsenoside Rb1
Rg1	Ginsenoside Rg1
OGD/R	Oxygen–glucose deprivation/reoxygenation
ROS	Reactive oxygen species (ROS)
CAT	Catalase
NADPH	Nicotinamide adenine dinucleotide phosphate
MMP	Mitochondrial membrane potential
MPTP	Mitochondrial permeability transition pore
mtDNA	Mitochondrial DNA
MRC	Mitochondrial respiratory chain
ATP	Adenosine triphosphate
OXPHOS	Oxidative phosphorylation
ETC	Electron transport chain
DCFA-DA	2′,7′-dichlorodihydrofluorescein diacetate
